# A 1-Month Ketogenic Diet Increased Mitochondrial Mass in Red Gastrocnemius Muscle, but Not in the Brain or Liver of Middle-Aged Mice

**DOI:** 10.3390/nu13082533

**Published:** 2021-07-24

**Authors:** Zeyu Zhou, Jocelyn Vidales, José A. González-Reyes, Bradley Shibata, Keith Baar, Jennifer M. Rutkowsky, Jon J. Ramsey

**Affiliations:** 1 Department of Molecular Biosciences, School of Veterinary Medicine, University of California, Davis, CA 95616, USA; zezhou@ucdavis.edu (Z.Z.); jrvidales@ucdavis.edu (J.V.); 2 Department of Cell Biology, Physiology and Immunology, Campus de Excelencia Internacional Agroalimentario, CeiA3, University of Córdoba, 14071 Córdoba, Spain; bc1gorej@uco.es; 3 Department of Cell Biology and Human Anatomy, School of Medicine, University of California, Davis, CA 95616, USA; bsshibata@ucdavis.edu; 4 Department of Neurobiology, Physiology, and Behavior, University of California, Davis, CA 95616, USA; kbaar@ucdavis.edu

**Keywords:** ketogenic diet, mitochondria, skeletal muscle, brain, liver

## Abstract

Alterations in markers of mitochondrial content with ketogenic diets (KD) have been reported in tissues of rodents, but morphological quantification of mitochondrial mass using transmission electron microscopy (TEM), the gold standard for mitochondrial quantification, is needed to further validate these findings and look at specific regions of interest within a tissue. In this study, red gastrocnemius muscle, the prefrontal cortex, the hippocampus, and the liver left lobe were used to investigate the impact of a 1-month KD on mitochondrial content in healthy middle-aged mice. The results showed that in red gastrocnemius muscle, the fractional area of both subsarcolemmal (SSM) and intermyofibrillar (IMM) mitochondria was increased, and this was driven by an increase in the number of mitochondria. Mitochondrial fractional area or number was not altered in the liver, prefrontal cortex, or hippocampus following 1 month of a KD. These results demonstrate tissue-specific changes in mitochondrial mass with a short-term KD and highlight the need to study different muscle groups or tissue regions with TEM to thoroughly determine the effects of a KD on mitochondrial mass.

## 1. Introduction

An increase in mitochondrial mass has been proposed as one underlying mechanism for the therapeutic and health-promoting effects of ketogenic diets (KD) [1,2]. Changes in levels of mitochondrial markers in skeletal muscle [3,4,5,6], brain [5,7], and liver [5,6,8,9] have been observed in some rodent models with KDs. However, most studies used only a few markers (e.g., citrate synthase or mitochondrial DNA), which give an incomplete picture of mitochondrial changes induced by diet as each marker has its limitations, possibly leading to the discrepancies observed among studies. We have previously used a panel of common mitochondrial markers to thoroughly investigate the impact of a KD started in early middle-aged mice on mitochondrial mass in whole-tissue homogenates of hindlimb skeletal muscle, brain, and liver, and after 1 month of intervention, no concerted changes in markers of mitochondrial mass were observed [10]. However, it is not known whether mitochondrial mass in specific muscle groups or regions within a tissue may be altered with a KD. For example, skeletal muscle mitochondrial mass varies depending on fiber type, and muscle regions that are denser in oxidative fibers (type I and IIa) may be impacted by a shift in fuel utilization to a greater extent than other muscle groups. In support of this hypothesis, we have previously demonstrated that prolonged KD preferentially preserved oxidative muscle fibers. In particular, a shift from type IIb to oxidative type IIa fibers was observed with a KD [11]. Transmission electron microscopy (TEM) is considered the gold standard for mitochondrial quantification in tissues [12]. Thus, TEM is needed to further unravel possible KD-induced changes in mitochondrial mass, and the use of TEM also allows the selection of specific regions of interest within a tissue.

Morphological quantification of mitochondrial content using TEM has not been performed in skeletal muscle or liver of healthy middle-aged rodents consuming a KD, and studies are needed using TEM to elucidate the actual changes in mitochondrial mass in these tissues. TEM has been utilized to quantify changes in mitochondrial content in brain regions with KDs in rodent models of neurological diseases [13,14,15], but little is known on how a KD affects mitochondrial mass in healthy adult rodents. A few studies have been conducted in aged rats to investigate effects of a medium chain triglyceride (MCT)-supplemented KD on mitochondrial content in the hippocampus and cerebellum [16,17,18]. However, changes in the prefrontal cortex, which are believed to mediate many of the cognitive declines seen with neurological disorders and aging [19], have not been studied.

The current study used TEM to perform morphological quantification of mitochondrial mass in regions of skeletal muscle, the liver, and the brain in middle-aged mice following 1 month of KD. Red gastrocnemius muscle was selected as the region of interest for skeletal muscle since this muscle group is widely studied for mitochondrial content and contains a variety of fiber types, including a relatively high level of oxidative fibers [20]. For the liver, the middle region of the largest liver lobe (the left lobe) was selected. Both the hippocampus and prefrontal cortex were selected for brain since these regions are heavily involved in memory and are susceptible to changes with aging.

## 2. Materials and Methods

### 2.1. Animals and Diets

Male C57BL/6JN mice were obtained at 11 months of age from the NIA Aged Rodent Colony. The housing conditions and diets were the same as described in Roberts et al. [21]. Mice were randomly assigned to a control (CD) or ketogenic diet (KD), with body weight counterbalanced in each group, at 12 months of age and were fed 11.2 kcal/day throughout the study. The CD contained (% of total kcal) 18% protein, 65% carbohydrate, and 17% fat. The KD contained 10% protein, <1% carbohydrate, and 89% fat. After 1 month of intervention, or at 13 months of age, animals were euthanized with CO_2_ inhalation in the morning following an overnight fast. All animal protocols were approved by the UC Davis Institutional Animal Care and Use Committee (protocol number 20054) and were in accordance with the NIH guidelines for the Care and Use of Laboratory Animals. Body weight was not significantly different between CD and KD mice at the end of the study (CD: 31.85 ± 1.16 g, KD: 32.07 ± 0.52 g).

### 2.2. Tissue Processing for Transmission Electron Microscopy

The left liver lobe, dorsal hippocampus, prefrontal cortex, and red gastrocnemius were selected as representative regions for the liver, brain, and skeletal muscle. Tissues were quickly dissected and cut into approximately 1 mm^3^ cubes in ice-cold PBS. Samples were fixed in 2.5% glutaraldhyde and 2% paraformaldehyde in 0.1M sodium phosphate buffer overnight, then rinsed in 0.1M sodium phosphate twice for 15 min. Tissues were post-fixed in 1% osmium tetroxide. After rinsing in distilled water, samples were dehydrated with a graded ethanol series from 50–100%. The samples were suspended in propylene oxide twice for 15 min and then pre-infiltrated overnight in 1:1 propylene oxide: resin (Dodecenyl Succinic Anhydride, Araldite 6005, Epon 812, Dibutyl Phthalate, Benzyldimethylamine) followed by infiltrating for 5 h in 100% resin. The samples were embedded in fresh resin and polymerized for 24 h at 70 °C. Approximately 100 nm sections were cut using a Leica EM UC6 ultramicrotome and collected on copper grids. The sections were stained with 4% aqueous uranyl acetate followed by 0.3% lead citrate in 0.1N sodium hydroxide and imaged with a FEI Talos L120C transmission electron microscope (Thermo Fisher, Waltham, MA, USA).

### 2.3. Micrograph Processing and Mitochondrial Mass Quantification

**Figure 1:**
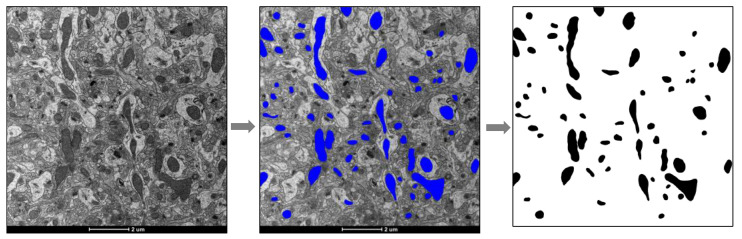
Micrographs were processed and analyzed using Image J (NIH) to calculate mitochondrial fractional area.

For the hippocampus and prefrontal cortex, 10 images were randomly taken for each mouse (*n* = 5–6) at 5300× magnification and only regions of neuropils were included. For red gastrocnemius muscle, 10 images each were taken in the subsarcolemmal (SSM) and intermyofibrillar mitochondrial (IMM) regions from 3–5 fibers for each mouse (*n* = 4–5) at 5300× magnification. Images of whole hepatocytes were taken at 1600–2600× magnification, and 4–6 cells were used for each mouse (*n* = 6). The images were then processed through Image J (NIH). The outline of each mitochondrion was precisely drawn using a Surface Pro 6 tablet equipped with a touch pen (Microsoft, Redmond, WA, USA), and filled with a solid bright color. The image with color-filled mitochondria was converted into a binary black and white image (Figure 1) using the “color threshold” command, and then areas of mitochondria were generated using the “analyze particles” command. Briefly, this command recognized the black objects (mitochondria) in the binary images and outlined them such that the area of each outlined object was automatically computed. Fractional area was calculated as total mitochondrial area divided by image area for the brain and muscle, and cytosolic area for the liver. Areas of lipid droplets in hepatocytes were measured, and mitochondrial fractional area was calculated with lipid droplets included or excluded (data not shown), and the results did not significantly differ using either approach. Mitochondrial number and average mitochondrial area (an indication of mitochondrial size) were also measured.

### 2.4. Statistical Analysis

All values are expressed as mean ± SEM unless otherwise indicated. Unpaired *t*-tests were performed to compare mitochondrial fractional area, density, and average size measured by TEM between groups. Significance for all comparisons was set at *p* < 0.05. All statistical analyses were conducted using GraphPad Prism 8.1 (GraphPad Software Inc., San Diego, CA, USA).

## 3. Results

### 3.1. One Month of a KD Increased Both Subsarcolemmal (SSM) and Intermyofibrillar (IMM) Mitochondrial Mass in Red Gastrocnemius Muscle

**Figure 2:**
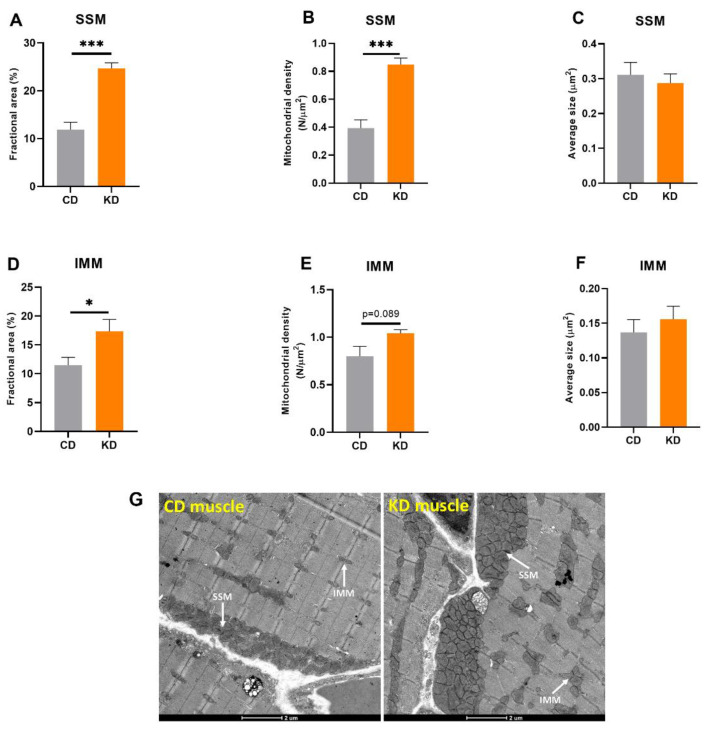
Quantification of subsarcolemmal (SSM) and intermyofibrillar (IMM) mitochondrial mass in red gastrocnemius muscle (*n* = 4–5 mice, 10 images per mouse). Fractional area of (**A**) SSM and (**D**) IMM. Number density of (**B**) SSM and (**E**) IMM per micrograph. Average mitochondrial size of (**C**) SSM and (**F**) IMM. (**G**) Representative micrographs of red gastrocnemius muscle of CD and KD animals. Diets: CD = control, KD = Ketogenic. * *p* < 0.05, *** *p* < 0.001.

To investigate mitochondrial mass in a specific hindlimb muscle, TEM analysis was completed in red gastrocnemius muscle after 1 month of a KD. Results from TEM analysis showed both SSM and IMM fractional area was significantly higher in KD mice (Figure 2A,D), consistent with an increase in mitochondrial content, and this appeared to be driven by an increase in the number of mitochondria in both regions (Figure 2B,E). There was no difference in the average size of mitochondria between diet groups (Figure 2C,F).

### 3.2. Mitochondrial Mass Was Not Altered in Prefrontal Cortex or Hippocampus after 1 Month of a KD

**Figure 3:**
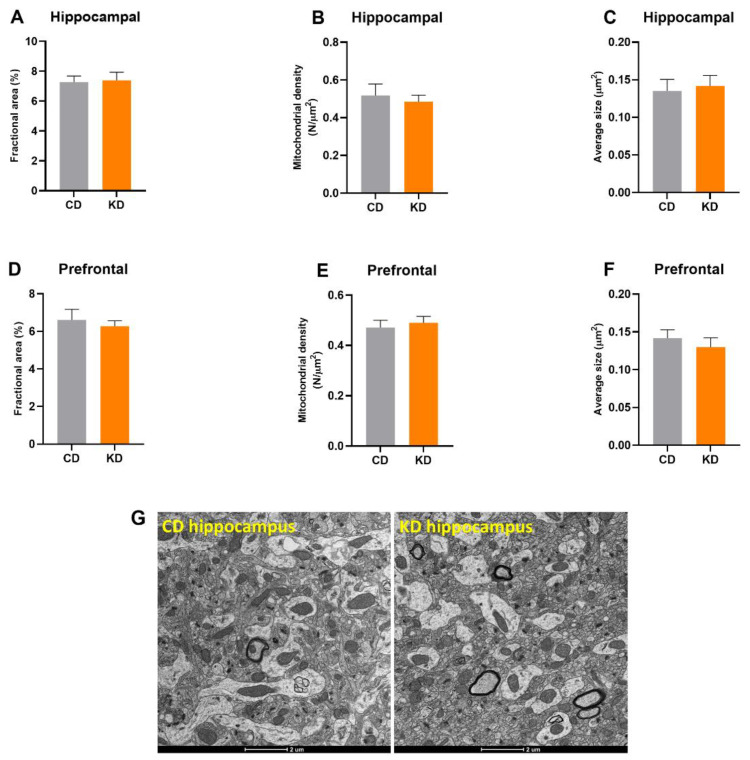
Quantification of mitochondrial mass in the prefrontal cortex and dorsal hippocampus (*n* = 5–6 mice, 10 images per mouse). Fractional area of (**A**) hippocampal and (**D**) prefrontal mitochondria. Number density of (**B**) hippocampal and (**E**) prefrontal mitochondria per micrograph. The average size of (**C**) hippocampal and (**F**) prefrontal mitochondria. (**G**) Representative brain micrographs of CD and KD animals. Diets: CD = control, KD = Ketogenic.

Mitochondrial quantification using TEM did not show any differences in prefrontal cortex or hippocampus fractional area after 1 month of a KD (Figure 3A,D). Similarly, mitochondrial density and average mitochondrial size were not significantly altered by diet (Figure 3B,C,E,F).

### 3.3. Hepatic Mitochondrial Mass Was Not Altered after 1 Month of KD

**Figure 4:**
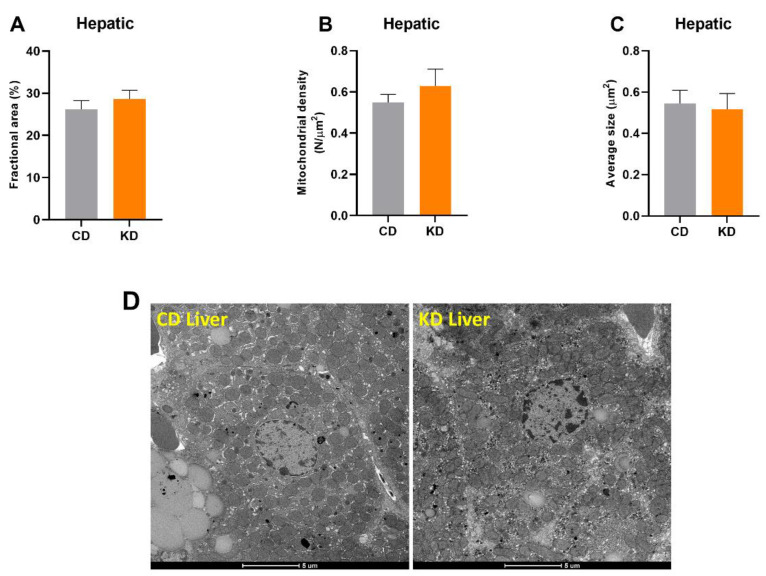
Quantification of mitochondrial mass in hepatocytes (*n* = 6 mice, 4–6 hepatocytes per mouse). (**A**) Fractional area (area of lipid droplets included), (**B**) number density, and (**C**) average size of hepatic mitochondria. (**D**) Representative hepatocyte micrographs of CD and KD animals. Diets: CD = control, KD = Ketogenic. All values are presented as mean ± SEM.

There was no change in mitochondrial area or number in hepatocytes measured with TEM after 1 month of KD (Figure 4A–C). No significant differences were observed between diet groups either before or after adjustment for lipid droplet area.

## 4. Discussion

The goal of this study was to quantify mitochondrial content in regions of skeletal muscle, brain, and liver following one month of a KD using the gold standard, TEM. Our data indicate that one month of a KD results in increased mitochondrial mass in red gastrocnemius muscle, but not the liver or brain.

In our previous skeletal muscle work [10], mitochondrial markers were analyzed in homogenates of the entire hindlimb skeletal muscle, and it was not possible to determine the response of specific muscles to a KD. To directly address this issue, we harvested the red gastrocnemius muscle to measure mitochondrial content with TEM. The TEM results demonstrated that 1 month of a KD increased both SSM and IMM content in red gastrocnemius muscle. Aging is associated with a reduction in mitochondrial content in oxidative muscle fibers, and preservation of mitochondrial mass is thought to alleviate the age-related decline in muscle function and strength [11,22]. The increase in red gastrocnemius muscle mitochondrial mass may contribute to the previous observation that aged mice on a KD perform better in muscle strength and endurance tests than CD animals [21]. However, these changes in mitochondrial mass were not reflected in the hindlimb skeletal muscle mitochondrial markers assayed at this same length of time on diet in our previous study [10]. This could indicate the markers used were not sensitive enough to pick up the differences in muscle tissues. However, we think this is unlikely since previous studies have found good correlations between several of these markers and TEM measurements [12]. Instead, the TEM results likely indicate that KD-related increases in mitochondrial content in red gastrocnemius do not translate to other muscles. In our previous work, we pooled all of the muscle of the hindlimb for analysis [10], and the likely result was the greater mass of glycolytic muscles obscured the rapid change in mitochondria in the smaller oxidative muscle mass when whole hindlimb homogenates were used to study skeletal muscle. In support of this hypothesis, our recent study [11] looking specifically at the gastrocnemius muscle following two months of a KD in middle-aged mice demonstrated upregulated mitochondrial biogenesis and increased citrate synthase activity. These results highlight the need for measurements in multiple muscle groups to more thoroughly determine the impact of KDs on skeletal muscles.

In the present study, no changes in mitochondrial mass quantification by TEM were observed between diet groups in the hippocampus or prefrontal cortex with a KD. These data are consistent with our previous finding that markers of mitochondrial mass were not altered in brain after one month of a KD. With a long-term KD of 14 months, most markers of mitochondrial content were not altered either, although citrate synthase activity did increase [10]. Balietti and colleagues showed similar results in that an 8-week MCT-supplemented KD had no impact on fractional mitochondrial volume in the hippocampus [18] and regions of the cerebellum [16,17] of 21-month-old rats compared to aged-matched control animals. In contrast, Bough and colleagues reported that in rats 22 days on a KD increased mitochondrial number in the hippocampus [23]. It should be noted this study used very young animals (P37–41), and the KD could have different effects on mitochondrial content in growing versus adult animals. Moreover, the KD animals were calorie restricted and had a significantly lower body weight compared to the control animals, and it is possible that calorie restriction may have played a role in the changes observed in this study. A few studies in mouse models of neurological disorders have shown that a KD increased hippocampal mitochondrial quantity [13,14]. Furthermore, an 8-week study in a mouse model of glaucoma found a KD increased mitochondrial number and area in retinal ganglion cell axons [15]. These studies suggest that a KD may increase mitochondrial content under certain conditions in some neural populations. However, in healthy middle-aged mice, a KD does not increase mitochondrial content in the prefrontal cortex or hippocampus.

In the liver, mitochondrial enzyme markers, especially citrate synthase, can be affected by shifts in metabolism and induction of gluconeogenesis with a KD [10] and as a result may be poor markers of mitochondrial mass in the liver. Thus, use of TEM to quantify mitochondrial content is important to determine the effects of a KD on mitochondrial mass in the liver. The results in the liver are consistent with our previous observation that markers other than mitochondrial enzymes (e.g., cardiolipin) were not changed after one month of a KD [10]. Eagles et al. showed that lipid droplets were increased in the liver of rats fed a KD [24]. However, these authors used a KD diet that was deficient in choline, which could lead to liver steatosis with a high-fat diet [25]. The present study did not observe a change in lipid droplets in hepatocytes with a KD (data not shown), and mitochondrial fractional area did not differ between CD and KD animals before or after adjusting for areas of lipid droplets.

## 5. Conclusions

Collectively, the results of the present study demonstrate that one month of a KD increased mitochondrial mass in red gastrocnemius muscle but had no impact on mitochondrial content in the liver, hippocampus, and prefrontal cortex. These results highlight the tissue-specific effects of a short-term KD on mitochondrial mass and the need for future measurements in specific muscle groups and different regions within a tissue to thoroughly determine the impact of KDs on mitochondrial mass.

## Data Availability

The data used to form the figures can be provided by the corresponding authors on request.
